# Comparison of hyperpolarized ^3^He-MRI, CT based parametric response mapping, and mucus scores in asthmatics

**DOI:** 10.3389/fphys.2023.1178339

**Published:** 2023-08-01

**Authors:** Katherine J. Carey, Peter Hotvedt, David G. Mummy, Kristine E. Lee, Loren C. Denlinger, Mark L. Schiebler, Ronald L. Sorkness, Nizar N. Jarjour, Charles R. Hatt, Craig J. Galban, Sean B. Fain

**Affiliations:** ^1^ Department of Medical Physics, University of Wisconsin—Madison, Madison, WI, United States; ^2^ Department of Radiology, University of Wisconsin—Madison, Madison, WI, United States; ^3^ Imbio LLC, Minneapolis, MN, United States; ^4^ Department of Nuclear Engineering, University of Michigan—Ann Arbor, Ann Arbor, MI, United States; ^5^ Center for In Vivo Microscopy, Department of Radiology, Duke University, Durham, NC, United States; ^6^ Center for In Vivo Microscopy, Duke University, Durham, NC, United States; ^7^ Department of Biostatistics, University of Wisconsin—Madison, Madison, WI, United States; ^8^ Division of Allergy, Pulmonary, and Critical Care Medicine, University of Wisconsin—Madison, Madison, WI, United States; ^9^ School of Pharmacy, University of Wisconsin—Madison, Madison, WI, United States; ^10^ Department of Radiology, University of Michigan, Ann Arbor, MI, United States; ^11^ Department of Radiology, University of Iowa, Iowa City, IA, United States

**Keywords:** asthma, MRI, CT, gas trapping, hyperpolarized He-3, parametric response mapping (PRM), ventilation

## Abstract

**Purpose:** The purpose of this study was to anatomically correlate ventilation defects with regions of air trapping by whole lung, lung lobe, and airway segment in the context of airway mucus plugging in asthma.

**Methods:** A total of 34 asthmatics [13M:21F, 13 mild/moderate, median age (range) of 49.5 (36.8—53.3) years and 21 severe, 56.1 (47.1—62.6) years] and 4 healthy subjects [1M:3F, 38.5 (26.6—52.2) years] underwent HP ^3^He MRI and CT imaging. HP ^3^He MRI was assessed for ventilation defects using a semi-automated k-means clustering algorithm. Inspiratory and expiratory CTs were analyzed using parametric response mapping (PRM) to quantify markers of emphysema and functional small airways disease (fSAD). Segmental and lobar lung masks were obtained from CT and registered to HP ^3^He MRI in order to localize ventilation defect percent (VDP), at the lobar and segmental level, to regions of fSAD and mucus plugging. Spearman’s correlation was utilized to compare biomarkers on a global and lobar level, and a multivariate analysis was conducted to predict segmental fSAD given segmental VDP (sVDP) and mucus score as variables in order to further understand the functional relationships between regional measures of obstruction.

**Results:** On a global level, fSAD was correlated with whole lung VDP (*r* = 0.65, *p* < 0.001), mucus score (*r* = 0.55, *p* < 0.01), and moderately correlated (−0.60 ≤ r ≤ −0.56, *p* < 0.001) to percent predicted (%p) FEV1, FEF25-75 and FEV1/FVC, and more weakly correlated to FVC%p (−0.38 ≤ *r* ≤ −0.35, *p* < 0.001) as expected from previous work. On a regional level, lobar VDP, mucus scores, and fSAD were also moderately correlated (r from 0.45–0.66, *p* < 0.01). For segmental colocalization, the model of best fit was a piecewise quadratic model, which suggests that sVDP may be increasing due to local airway obstruction that does not manifest as fSAD until more extensive disease is present. sVDP was more sensitive to the presence of a mucus plugs overall, but the prediction of fSAD using multivariate regression showed an interaction in the presence of a mucus plugs when sVDP was between 4% and 10% (*p* < 0.001).

**Conclusion:** This multi-modality study in asthma confirmed that areas of ventilation defects are spatially correlated with air trapping at the level of the airway segment and suggests VDP and fSAD are sensitive to specific sources of airway obstruction in asthma, including mucus plugs.

## 1 Introduction

Severe asthma exhibits considerable heterogeneity both across patients and within the lungs of an individual patient. Ventilation heterogeneity, or the non-uniform distribution of inspired gas within the lungs is a feature of asthma revealed by ventilation defects observed on HP gas MRI (Downie et al., 2007; Teague et al., 2014). Areas of ventilation defects have previously been shown to be associated with mucus plugging of the central airway as visualized on CT (Svenningsen et al., 2019; Mummy et al., 2022), areas of low density lung parenchyma on CT, thought to represent regions of air trapping (Fain et al., 2008), and local airway wall thickening presumably due to remodeling (Svenningsen et al., 2014). The ventilation defect percent, or VDP, has been established as a marker of asthma instability (Mummy et al., 2020). VDP has been extended to a regional measure using deformable registration to CT lobar and segmental structures, i.e., segmental VDP (sVDP), to guide bronchial ablation therapy (Thomen et al., 2015) and to better quantify regional association of VDP and mucus plugging (Mummy et al., 2022). However, the etiology of ventilation defects and air trapping in asthma are still under investigation.

Parametric response mapping (PRM) is a technique used to classify lung tissue based on densitometry at both inspiration and expiration (Galban et al., 2012) with the advantage of isolating inspiratory, i.e., emphysema, from expiratory low density lung parenchyma. The percent of low density lung parenchyma at expiratory lung volume from PRM corresponds to the functional small airways disease (fSAD) and has been shown to be associated with future spirometry decline in COPD (Bhatt et al., 2016) and asthma (Krings et al., 2021). fSAD is identical to air trapping measured using the −856 HU threshold (Lynch and Al-Qaisi, 2013) in asthma since emphysema is not a major component of the measurement.

The regional overlap of ventilation defects with air trapping is expected from previous work (Fain et al., 2008), but the anatomic co-localization at the lobar and airway segment level has not been directly studied. New measures of regional obstruction, including sVDP and mucus score, can provide anatomically precise co-localization of obstructive measures. This makes it possible to test spatial associations of ventilation defects with air trapping and their interaction with mucus plugging to gain insights into their functional significance in asthma. Here, we evaluate global and regional correlations between VDP and sVDP on HP ^3^He MRI with mucus plugging and fSAD on quantitative CT after administration of four puffs of albuterol, a β-agonist bronchodilator (BD). The comparisons at the airway segment level in this work are the first quantitative co-localization of small airways and ventilation markers.

## 2 Materials and methods

### 2.1 Study population

Our study population was drawn from the National Heart, Lung, and Blood Institute (NHLBI) Severe Asthma Research Program III (SARP3) population (Teague et al., 2018) recruited and imaged between 2012 and 2016. The study was compliant with the Health Insurance Portability and Accountability Act (HIPAA) and approved by the Institutional Review Board (IRB). Written informed consent was obtained from all subjects. The population was divided into mild/moderate and severe asthma groups as defined by the SARP criteria (Jarjour et al., 2012). The HP ^3^He MRI studies were conducted under Food and Drug Administration (FDA) investigational new drug (IND) protocol #064867. An overview of the study population and procedures is shown in Table 1.

**TABLE 1:** Study population and image processing steps with number and percent of total participants at each stage. All imaging is post-bronchodilator. Percentages in the Healthy and Asthmatics columns are relative to the starting population; percentages in the Severe column are relative to the corresponding population at that stage.

Image acquisition and processing stages	Healthy N (%)	Total asthmatics (severe + moderate) N (%)	Severe N (%)
1. Total SARPIII asthmatic population at UW-Madison	5 (100%)	100 (100%)	62 (62.0%)
2. Subjects with HP ^3^He MRI	5 (100%)	78 (78%)	48 (61.5%)
3. With inspiratory and expiratory CT	4 (80%)	34 (34%)	21 (61.7%)
4. With scored segmental mucus plugs	4 (80%)	34 (30%)	21 (61.7%)

CT, proton MRI, HP ^3^He MRI, and spirometry were all acquired on the same day. To mitigate the effects of airway hyperresponsiveness, all imaging and spirometry were acquired after administration of four puffs of albuterol, a β-agonist BD. Percent Predicted (PP) values for forced expiratory volume in one second (FEV_1_), forced vital capacity (FVC), and FEV_1_/FVC were generated using the Global Lung Function Initiative reference values (Krings et al., 2021).

### 2.2 Imaging methods

Volumetric multidetector CT (MDCT) was acquired post-BD at both total lung capacity (TLC) and functional residual capacity (FRC) each during a breath hold of approximately 4 s using a GE Light Speed CT scanner with 64 detectors (0.625 mm^2^ voxel size in-plane, 0.5 mm slice thickness). Images were reconstructed using a standard kernel. Specific acquisition parameters are presented in Supplementary Table S1.

MRI was acquired using a 1.5T Signa HDx GE scanner (GE Healthcare, Milwaukee, WI) with either a flexible (IGC Medical Advances, Milwaukee, WI) or a rigid-body (Rapid Biomedical, Columbus, OH) single-channel volume coil, depending on patient size. Both coils were tuned to operate at the resonant frequency of ^3^He and decoupled from the body RF coil so that proton MRI and HP ^3^He MRI could be acquired consecutively without moving the subject during matched breath-hold inflation volumes. The ^3^He studies in this work were conducted using Polarean IGI.9600 polarizer (Polarean Imaging plc, Durham, NC) and using the SEOP method described previously (Lynch and Al-Qaisi, 2013).

A 4.5-mM dose of HP ^3^He mixed with N_2_ normalized to 14% of the subject’s predicted total lung capacity (TLC) was prepared in a Tedlar™ bag (Jensen Inert Products, Coral Springs, FL) purged of oxygen to slow T1 relaxation. The subject was positioned supine in the scanner and inhaled the gas dose post-bronchodilator from functional residual capacity (FRC) through a short plastic tube attached to the bag. Subjects were instructed to hold their breath through a 16–20 s acquisition, and blood oxygen saturation was monitored continuously using a pulse oximeter to ensure safety during and after the anoxic breath-hold. Proton MRI was acquired to match volume and slice location after inhalation to an identical lung inflation volume. Specific MRI parameters are summarized in Supplementary Table S2.

### 2.3 Image analysis

#### 2.3.1 MRI analysis

Ventilation defects were classified on HP ^3^He MRI using a semi-automated algorithm to calculate whole lung VDP (Zha et al., 2016). Proton MRI was registered to HP ^3^He MRI using a 3D rigid registration algorithm implemented using ANTs (http://stnava.github.io/ANTs/). The lung boundary was segmented on HP ^3^He MRI with reference to the proton MRI. The whole lung VDP was then identified as a percentage of total lung volume using an adaptive k-means classifier (Zha et al., 2016). Adaptive k-means is similar to standard k-means except that it conducts two rounds of clustering. The first identifies the lowest signal intensities, and the second reclassifies the lowest cluster from the first round to identify the fully obstructed subset. We referenced the performance of this method to radiologist observers who manually identified defects and then tuned the threshold of the reclassification of the low signal voxels to match the manual result. See (Zha et al., 2016; He et al., 2019) for more details.

#### 2.3.2 CT analysis

PRM analysis was applied to paired inspiratory and expiratory CT scans as previously described (Galban et al., 2012). Briefly, inspiratory CT was deformably registered to expiratory CT and voxel-wise changes in Hounsfield unit (HU) values of lung density were used to determine areas of normal lung parenchyma, non-emphysematous air trapping referred to as functional small airway disease (fSAD), emphysema (Emph), and parenchymal disease. Inspiratory CT images were processed through commercial package (VIDA Diagnostics, Coralville, IA) to generate a segmental anatomical mask. PRM percentages by bronchopulmonary lobe and segment (lobar and segmental PRM, respectively) were determined by applying the CT segmental mask to the PRM maps and calculating percent within the anatomical volume.

Mucus plug scoring was performed on CT by expert radiologists (B.M.E., D.S.G., J.D.N.,S.K.N., and M.L.S.) as part of a multisite effort within the SARP3 study, using the system developed by Dunican et al. (2018). Each individual bronchopulmonary segment was scored based on input from two readers and classified as having a mucus plug when at least one of the two readers scored a plug as present.

### 2.4 Image registration

**FIGURE 1:**
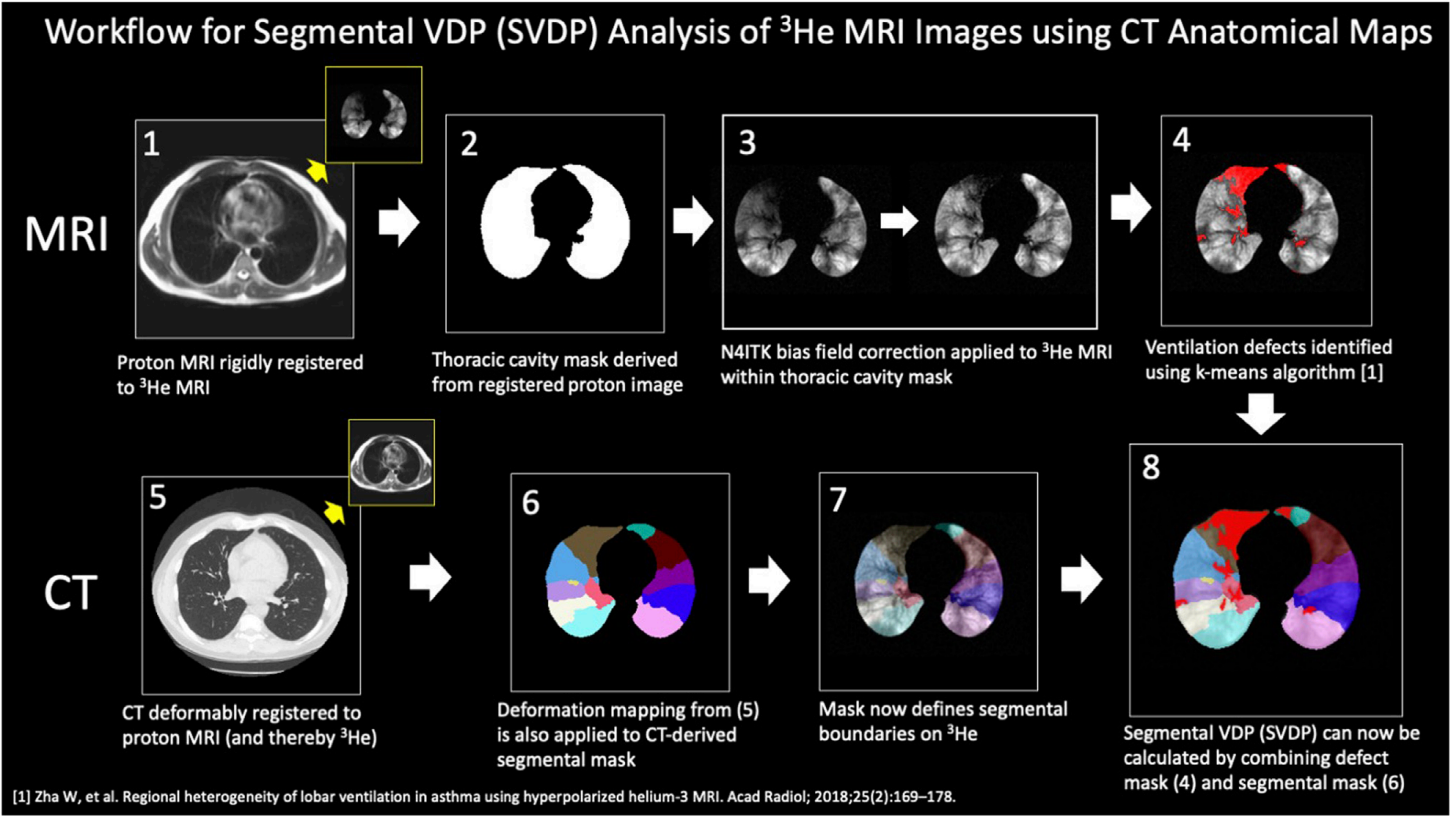
Steps in the analysis workflow to align segmental mask from CT to the ^3^He MRI ventilation images. The steps are as described in the body of the figure. The segmental VDP (sVDP) was then compared to the corresponding segmental airway fSAD from the parametric response map of the CT scan at the expiratory (FRC) lung volume. Note that the PRM map is inherently registered to CT and so can be readily compared by airway segment with ^3^He MRI after these analysis steps. Modified from Mummy et al. (2022). Used with permission.

As summarized in Figure 1, the CT lung boundary and segmental anatomical masks were generated using a commercial package (VIDA, Coralville, IA). To align to 3He MRI, the masks were then deformably registered to the anatomical proton MRI mask using the ANTs software package (http://stnava.github.io/ANTs/), and the resulting transformation was applied to the original CT images, thereby registering the segmental mask to the HP ^3^He MRI. VDP by individual bronchopulmonary segment (sVDP) were determined using the registered CT segmental mask registered to the whole lung ventilation defect mask, a method based on the technique described by *Thomen et al* (Thomen et al., 2015). The segmental VDP (sVDP) was then compared to the corresponding segmental airway fSAD from the parametric response map of the CT scan at the expiratory (FRC) lung volume. Note that the PRM map is inherently registered to CT and so can be readily compared by airway segment.

### 2.5 Statistical methods

Correlations between VDP, mucus plugging, and PRM measures on a whole lung and lobar level were calculated using the Spearman rank correlation. To further assess the spatial relationship between VDP, PRM_fSAD_, and mucus plugs, logarithmic values of segmental VDP and PRM_fSAD_ were used to build a multivariate linear mixed model to best compare relationships accounting for the different lung regions. Piecewise linear mixed models were fit to allow for separate estimation of the effects when sVDP = 0. When sVDP is greater than 0, we evaluated curvilinear models and found the quadratic model provided the best fit. The best (most parsimonious) model was selected based on the Akaike Information Criterion (AIC) and Likelihood Ratio tests (for nested models). Tests for interactions between sVDP and segment region were significant for the intercept but not the quadratic form. Similarly, interactions with mucus plugs were significant for intercept shifts but not for the quadratic form. A mixed effects heterogenous compound symmetry covariance structure was used (for expected symmetry between left and right lung segments). A *p*-value of 0.05 was used for the threshold of statistical significance. Whole lung and lobar statistical comparisons and segmental modeling were completed by the statistician on the project (K.L.).

## 3 Results

### 3.1 Study population

The study population consisted of 38 participants, including 4 healthy non-asthmatics, with 13 participants classified as having mild/moderate asthmatics and 21 as having severe asthma per the SARP3 criteria (Dunican et al., 2018). Population statistics are represented in Table 2. There were significant differences between Severe and Mild/Moderate Asthmatics with respect to BMI, FEV1 PP, FVC PP and VDP. By contrast, there were no significant differences between Severe and Mild/Moderate Asthma for fSAD, Emph, or Mucus Score. Of the 34 asthma patients, nine had mucus plugs. Those nine subjects had 80 segments with mucus plugs (about 14% of all segments had a mucus plug).

**TABLE 2:** Population statistics split by asthma severity, and all subjects combined. Quantitative measures are presented as median [1st quartile—3rd quartile]. Whole lung mucus score presented is the sum of all segmental mucus scores. Measures are acquired after administration of four puffs of bronchodilator. All statistics were calculated using the Wilcoxon Rank Sum test with a significance threshold of *p* < 0.05. PP, percent predicted.

	Healthy	Mild/Moderate asthmatics	Severe asthmatics	All
**N**	4	13	21	38
Gender	1M:3F	5 M: 8F	8M: 13F	14M:24F
Age (years)	38.5 [26.6—52.2]	49.5 [36.8—53.3]	56.1 [47.1—62.6]	51.2 [45.6—61.0]
High Dose ICS	N/A	3	21	24
Daily OCS Dependent (Dose)	N/A	0	3 (4, 7, 8 mg)	3
Monoclonal Rx	N/A	1	4	5
BMI	25.4 **^** [24.0–27.0]	27.7 **†** [24.7—29.0]	32.4 ^**†** [282—34.5]	29.0 [26.3—34.1]
FEV1 PP	111 **^** [106—166]	87.8**†** [83.0—94.6]	77.8% **^†** [69.1—97.7]	86.5% [75.4—105]
FVC PP	109 **^** [101—118]	101 **†** [86.6—109]	86.7 **^†** [82.9—97.2]	93.6% [84.8—105]
FEV1/FVC PP	102 **^** [99.0—104]	96.2 [89.1—99.1]	91.1 **^** [82.3—98.5]	96.1% [85.5—99.0]
FEF25-75 PP	118 *^ [113—121]	79.5* [61.8—95.2]	55.8 ^ [38.7—90.8]	78.0 [51.1—105]
VDP	0.275% *^ [0.0750—0.629]	2.51% *† [1.17—4.30]	6.18% **^†** [3.40—11.4]	3.43% [2.05—6.97]
fSAD	0.122% [0.0554–1.55]	0.620% [0.406—3.86]	1.36% [0.481—5.77]	1.19% [0.280—5.18]
Emph	0.0571% [0.0447—0.0816]	0.0875% [0.0613—0.124]	0.676% [0.0409—0.154]	0.0673% [0.0472—0.135]
Whole lung Mucus Score	0.0 [0.0—0.0]	0.0 [0.0—0.25]	0.0 [0.0—4.0]	0.0 [0.0—1.6]

*Significantly different between Healthy and Mild/Moderate Asthmatics.

^Significantly different between Healthy and Severe Asthmatics.

^†^Significantly Different between Mild/Moderate and Severe Asthmatics.

### 3.2 Global analysis

**FIGURE 2:**
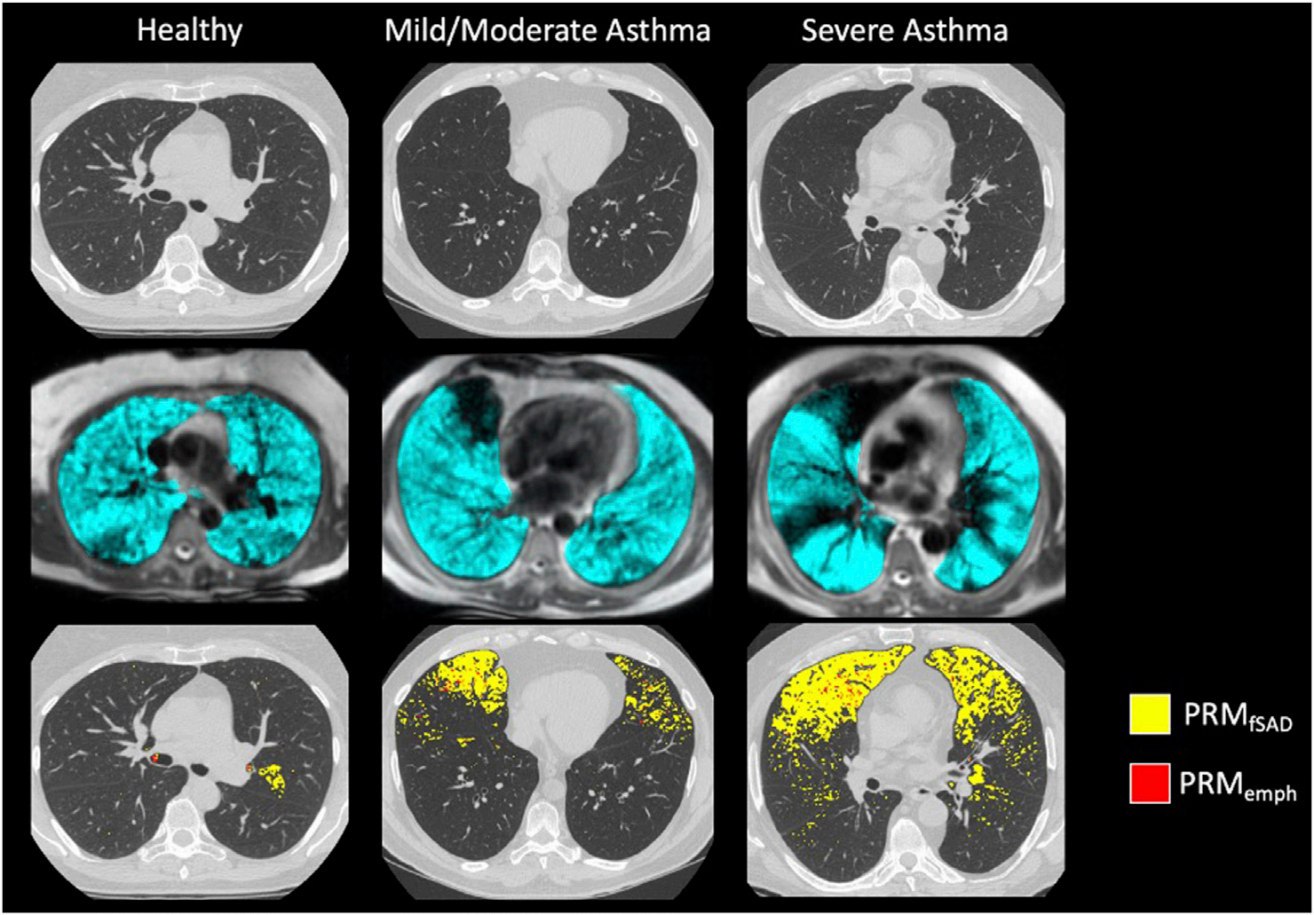
Example images of (from top to bottom): Inspiratory CT, HP 3He MRI overlaid on conventional MRI, and PRM maps overlaid on Inspiratory CT for a (from left to right) healthy subject, moderate asthmatic, and severe asthmatic.

**FIGURE 3:**
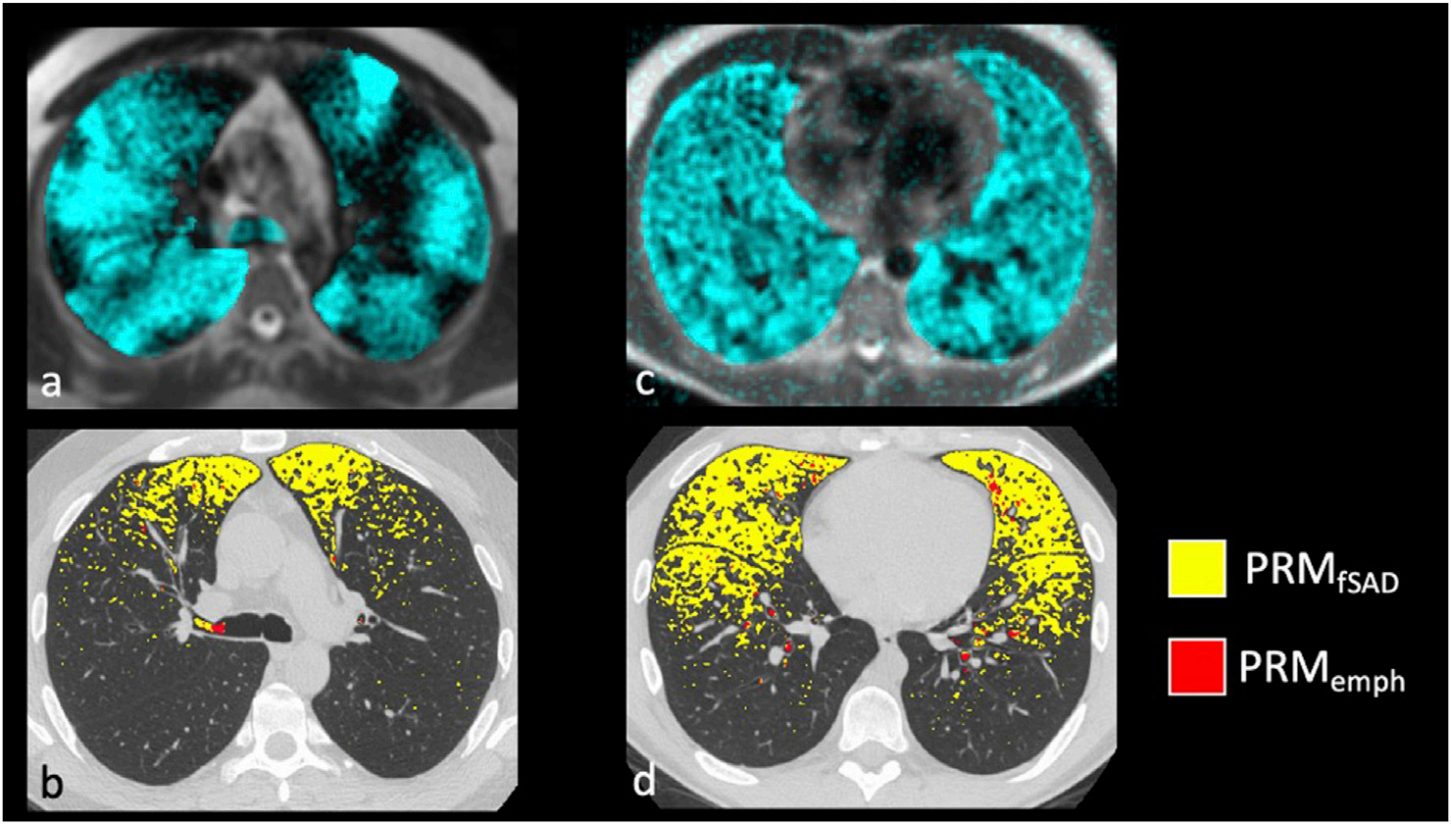
Examples of severe asthmatics where PRM and HP gas MRI are discordant. The most common discordant pattern is **(A,B)** HP 3He MRI ventilation > fSAD with whole lung VDP of 18.4% and PRMfSAD of 5.8%, and the least common is in **(C,D)** HP 3He MRI ventilation << fSAD with a whole lung VDP of 6.3% and PRMfSAD of 23.7%.

Confirming previous results, whole lung VDP and fSAD correlated with each other (Supplementary Figure S1), spirometry and mucus score (Supplementary Table S3). Qualitatively, larger areas of ventilation defect most commonly overlapped with areas of fSAD (Figure 2) but generally showed greater extent than fSAD (Figures 3A, B). More rarely, fSAD had greater extent, and in some participants, did not correspond to any local ventilation defect regions (Figures 3C, D).

### 3.3 Lobar analysis

**FIGURE 4:**
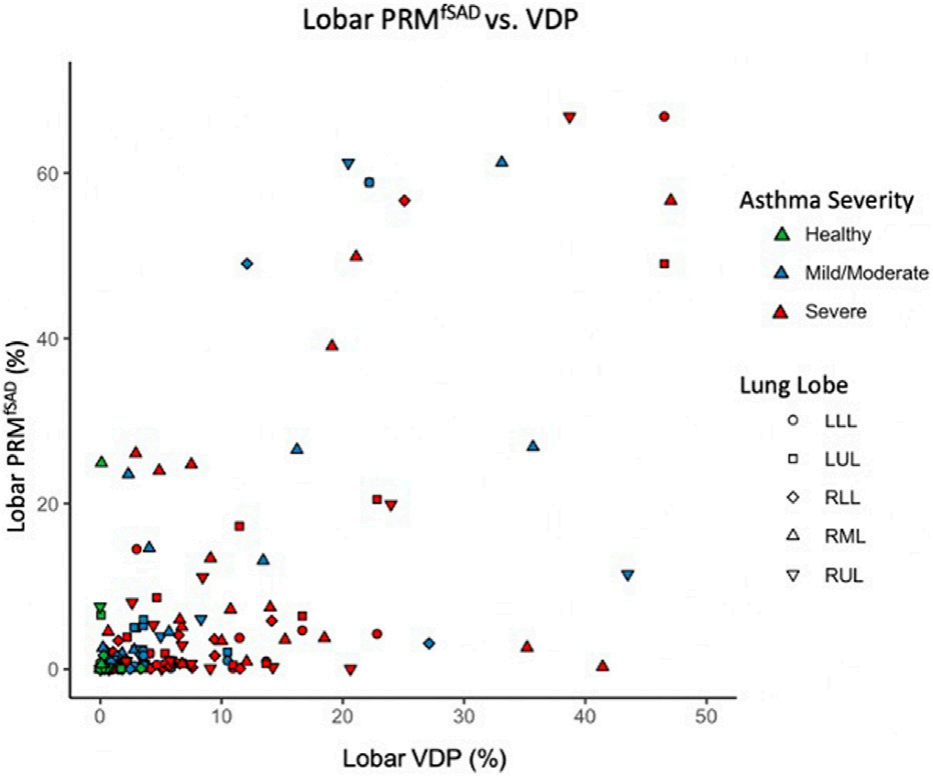
Scatter plot comparing lobar VDP to average whole lung and lobar fSAD. Points are colored based on disease severity and their shapes classify what lobe the datapoint is from. LLL, left lower lobe; LUL, left upper lobe; RLL, right lower lobe; RML, right middle lobe; RUL, right upper lobe.

Regional analysis comparing the extent of functional CT and MRI measures for the same lung lobe across all 38 participants (Table 3) showed that fSAD significantly correlated with lobar VDP in every lung lobe (r from 0.45–0.66, *p* < 0.01). Additionally, VDP correlated with mucus score in every lung lobe (r from 0.50–0.67, *p* < 0.01), while fSAD correlated with mucus score in 4 lobes (*r* = 0.38–0.47, *p* < 0.05) with the exception being the right lower lobe as shown in Table 3. A scatterplot comparing lobar fSAD and lobar VDP is shown in Figure 4.

**TABLE 3:** Spearman correlation coefficients by lobe for lobar fSAD compared to lobar VDP (left). Spearman correlation coefficients by lobe for lobar VDP and fSAD when compared to lobar mucus score (right). LUL, left upper lobe; LLL, left lower lobe; RUL, right upper lobe; RML, right middle lobe; RLL, right lower lobe. PP, Percent Predicted. Significance Key: *p < 0.05, **p < 0.01, ***p < 0.001.

	VDP	Lobar mucus score	
Lobe	fSAD	fSAD	VDP
LUL	0.59 ***	0.38 **	0.54 **
LLL	0.66 ***	0.47 **	0.50 **
RUL	0.52 **	0.44 *	0.67 ***
RML	0.51 **	0.47 **	0.62 ***
RLL	0.45 **	0.30	0.57 **

### 3.4 Segmental analysis

**FIGURE 5:**
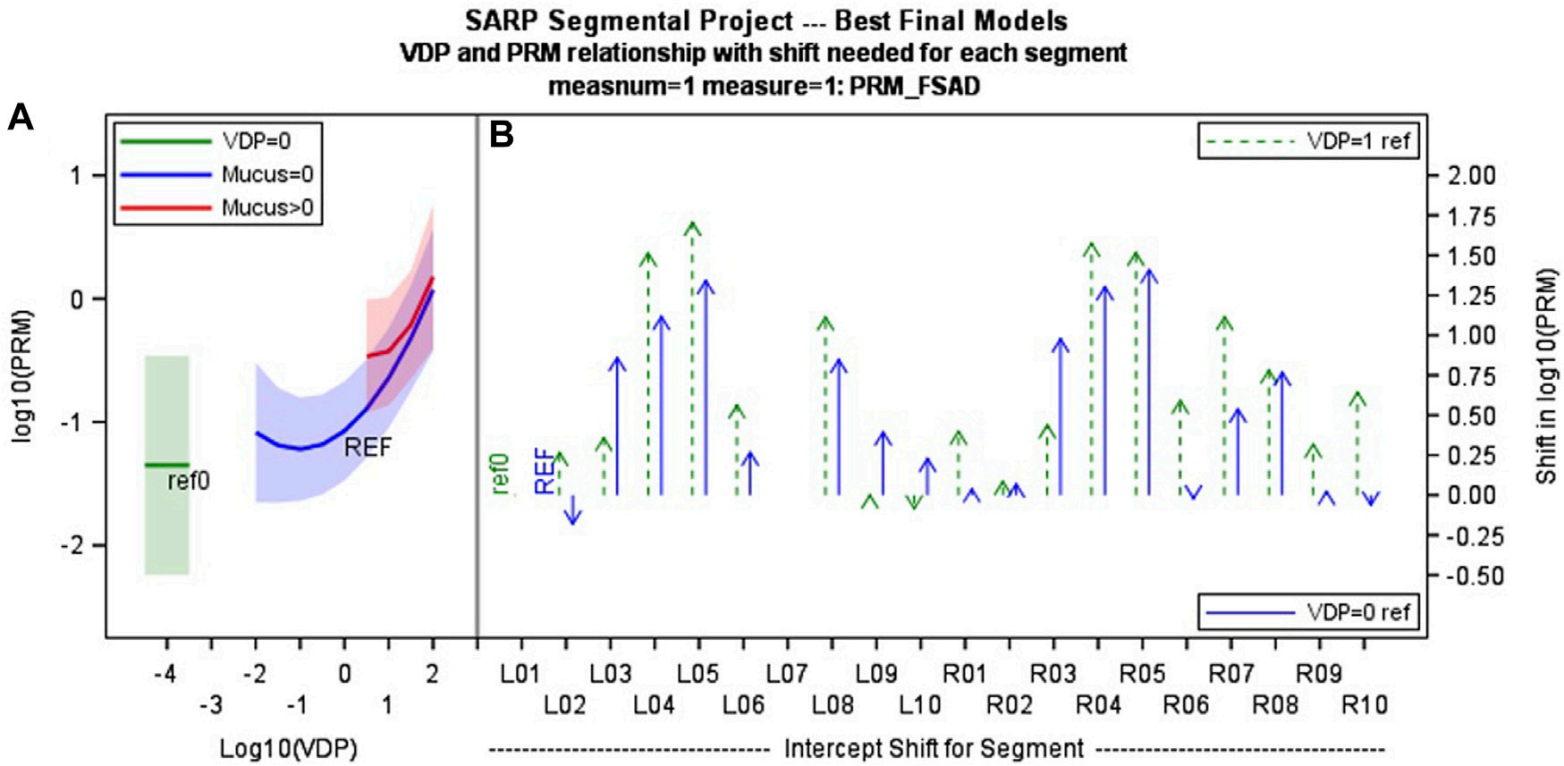
**(A)** shows the mixed effects piecewise multi-variate model showing how PRM fSAD changes with segmental VDP (with and without presence of mucus plug blue vs. red line). This functional relationship is common across all airway segments, but with **(B)** different intercepts for each segment (offsets relative to segmental VDP = 1 and segmental VDP = 0 are shown). These differences may relate to airway geometry, propensity for disease and/or inconsistency in lung inflation at functional residual capacity lung volume. **(C)** Segmental labels are defined as shown at far right. LB1 = Left bronchus segment 1, RB1 = right bronchus segment 1 etc.,… with index increasing from lung apex to base.

**FIGURE 6:**
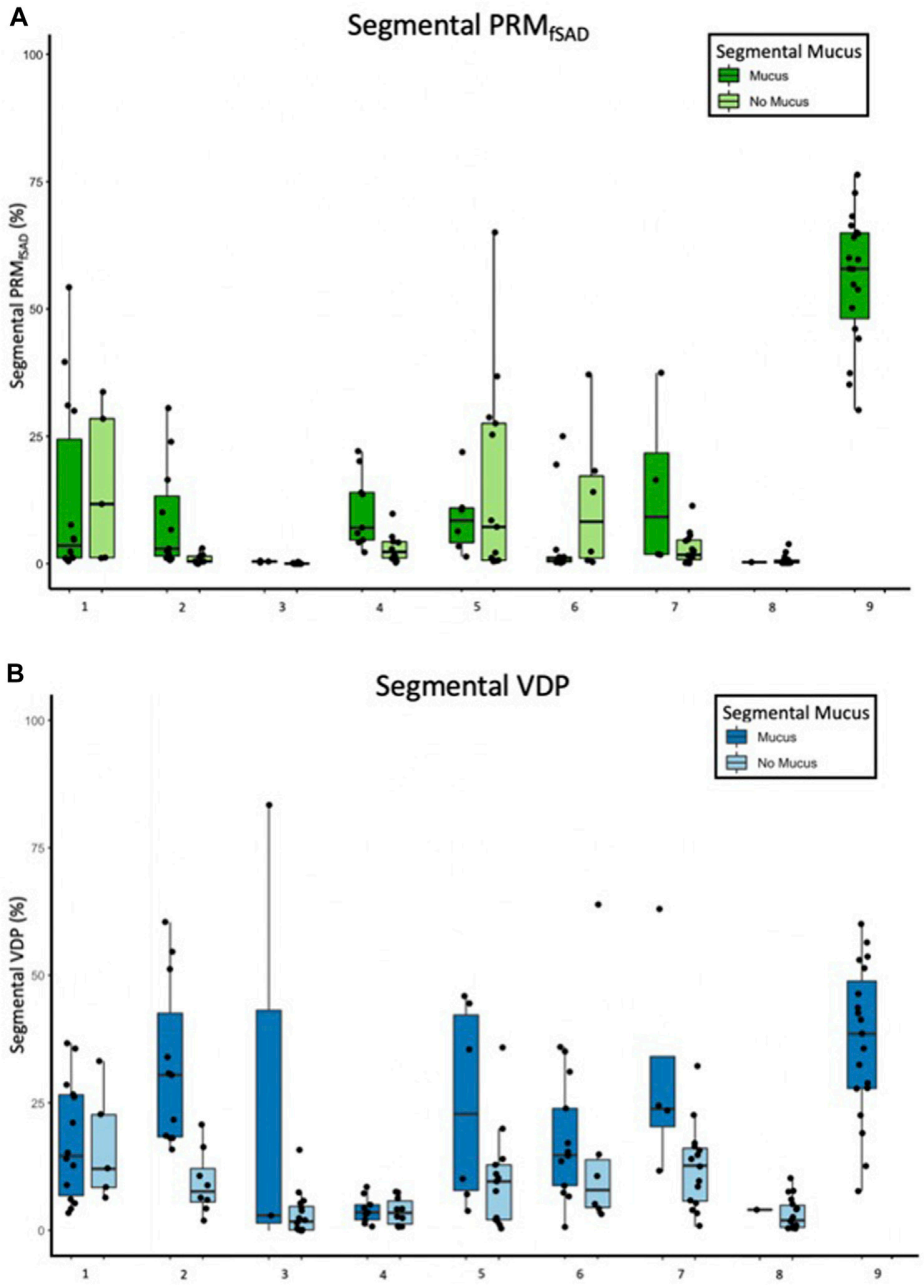
Box and whisker plots of **(A)** fSAD and **(B)** segmental VDP separated by segments with and without mucus plugs for each of 9 participants with non-zero mucus scores. Each data point is one bronchopulmonary segment and sums over both categories to the 19 segments analyzed.

**FIGURE 7:**
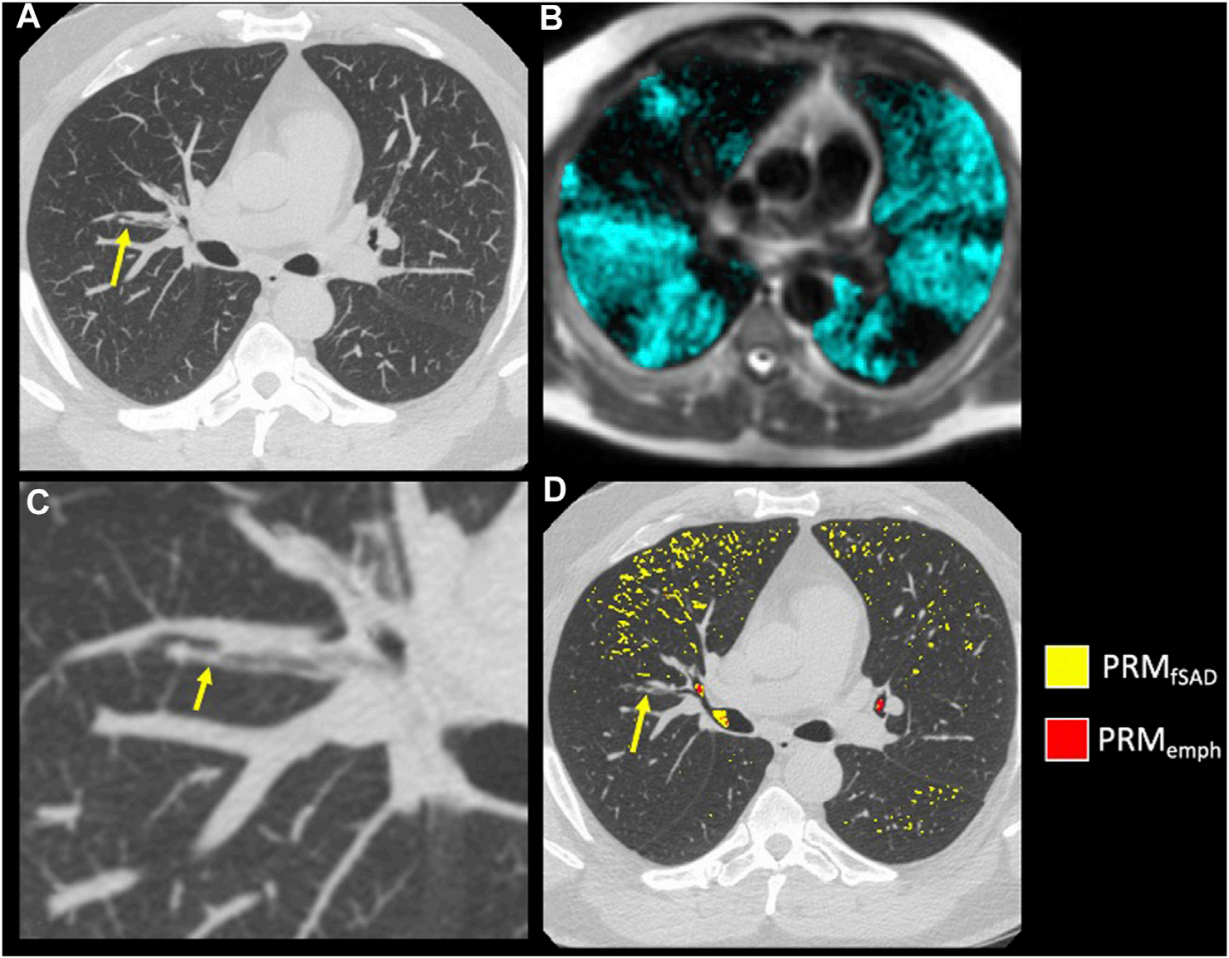
Example of mucus plugging within a moderate asthmatic. **(A)** A mucus plug (arrow) visualized on inspiratory CT using a MIP of 7 slices. **(B)** A close-up of the mucus plug (arrow) in **(A) (C)** HP 3He MRI overlaid on conventional MRI with ventilation defects occurring near and downstream of the mucus plug visualized in **(A)**. **(D)** PRM maps overlaid on Inspiratory CT in the axial slice of the mucus plug (arrow).

The model of best fit for comparing segmental fSAD as a function of sVDP was a piecewise quadratic model with an interaction with the presence of mucus plugs (Figure 5A) and separate intercepts for each airway segment. The expected fSAD when sVDP values are at or near zero (the intercept) varies across segments (Figure 5B). Measures of fSAD were largely constant or modestly positive linear with increasing moderate sVDP values, but the dependence increased markedly for higher VDPs, typically starting at or near sVDP >4% after which segmental fSAD increased with sVDP at a growing rate. Details on the statistical model are in Appendix B. The three pieces of the model (VDP = 0, VDP > 0, and presence of mucus plugs) are all significantly different from each other (*p* < 0.001). The differences in the presence of mucus plugs on fSAD (red line, Figure 5) are largely for moderate levels of sVDP between 4% and 10%—note that by the time sVDP is >10%, the difference in fSAD when mucus plugs are present is less prominent. Plots of segmental fSAD and sVDP in airway segments with and without mucus from our study cohort further support this finding, suggesting better contrast for detecting obstruction due to mucus on a per segment basis (Figures 6B vs. 6A) overall. To further illustrate, a qualitative example of this result is shown in Figure 7 where a mucus plug is visualized in a moderate asthmatic comparing the resulting HP gas MRI sVDP with the corresponding fSAD from PRM. The typical pattern shown is that sVDP is much greater than fSAD in the vicinity of the mucus plug.

## 4 Discussion

This is the first multimodality imaging study in patients with asthma to compare ventilation defects measured using the ventilation defect percent (VDP) with HP 3He MRI at the global, lobar and segmental level with functional small airways disease (fSAD) and airway mucus plugging after administration of four puffs of albuterol of BD. The fSAD is higher in segments with ventilation defects on HP 3He MRI. The fSAD measure, derived from parametric response mapping, identifies low density lung (−856 HU) on expiratory CT, and in asthma is synonymous with air trapping. The results corroborate previous work (Fain et al., 2008; Capaldi et al., 2016; Bell et al., 2019) showing correlation between ventilation heterogeneity and air trapping at the whole lung and qualitative regional levels (upper, middle, and lower and anterior/posterior). By comparing the measurements within physiologically defined anatomy of the lung, we show that VDP and fSAD are directly related at the lobar and segmental levels. The specific relationship was best described by a piecewise quadratic model with an interaction in the presence of airway mucus plugging suggesting fSAD responds to the presence of mucus plugging. Specifically, the interaction of fSAD with mucus plugs over a limited range of segmental VDP highlights potential differences in the sensitivity of these measures.

Importantly, the relationships between VDP and fSAD and their interactions with mucus plugs highlight potential differences in the measures that could be useful for interpretation. It was directly shown that fSAD is higher in segmental airway regions that also have ventilation defects, suggesting that the airway ventilation defects and air trapping likely share a similar etiology at the segmental level. However, it should be noted that spatial overlap was assumed in this analysis because the same segmental airway regions are shared but overlap was not directly measured. The interaction of fSAD’s dependence on VDP in the presence of a mucus plug shown in Figure 5 argues for a difference in degree of response that supports the idea that fSAD is emphasizing more distal vs. central airway obstruction. That is, the impact of mucus on fSAD is most pronounced at moderate levels of sVDP between 4% and 10%; this observation is consistent with fSAD being more sensitive to mucus plugging in the more distal small airways than sVDP since ventilation is only modestly reduced locally. By the time sVDP is >10%, the interaction with mucus between fSAD and sVDP is no longer apparent, which likely reflects the shared mechanisms of reduced gas delivery to the airway and its corresponding impact on gas retention leading to air trapping in that segment. Moreover, we are comparing a relatively low-resolution imaging modality, HP gas MRI, with results from a high-resolution imaging modality. fSAD derived from CT is likely more sensitive to changes on the scale of millimeters whereas HP gas MRI resolution is on the scale of a centimeter.

Despite the sensitivity of fSAD to early disease processes, we found that empirically, the sVDP better delineates visible mucus plugged segments on a per subject basis than fSAD. This is likely due to stochastic and inconsistency in lung inflation impacting lung density and airway closure, especially at functional residual capacity lung volume on the expiratory CT due to dose limitations (Appendix A) and inconsistencies in lung inflation that more greatly impact fSAD measurements (Boes et al., 2015). In our study CT scans were performed at functional residual capacity (FRC) rather than residual volume (RV), which may also contribute to physiologic variability in fSAD (Comellas et al., 2023). An advantage of VDP is that the signal is solely from direct gas filling of the airways from inhalation of the HP gas, which is more directly affected by central airway obstruction with consequent downstream opacification of the airway tree and is therefore less dependent on the lung inflation than densitometry on CT, although this needs further investigation.

Both fSAD and VDP are associated with future lung function decline and exacerbations in severe asthma (Mummy et al., 2020; Krings et al., 2021). To further establish the understanding of VDP and fSAD in asthma, additional studies are needed to investigate longitudinal changes in these metrics with special focus on regional measures and reducing sources of variability. VDP was highly correlated with measures of fSAD with similar functional dependence over each segment, but each segment had a different intercept. This suggests that airway anatomy is possibly pre-disposing each airway segment to more or less inconsistency in lung inflation at functional residual capacity lung volume and/or different levels of disease due to airway geometry or gravity dependence. An important area for future investigation is airway dysanapsis which may be an important factor in predisposing individuals and specific airway segments to more obstruction in asthma (Smith et al., 2020).

There are important limitations to this study. First, this study is small and representative of a single center. As asthma is a highly heterogenous disease, the relationship we observe between fSAD, mucus, and VDP may not be reflective of asthma in general. Second, the mucus score was performed independently by multiple radiologists involved in the SARP3 study and do not reflect a consensus read to resolve discordant scores. Similarly, the mucus score (Dunican et al., 2018) gives only a presence or absence measure in each segment so it is impossible to assess VDP and fSAD with respect to location or size of the mucus plug within a given airway segment. Third, the version of the parametric response mapping algorithm used in this work occasionally failed to fully remove airway regions from analysis, often near branch points of the central airways. These errors are a negligible fraction of the lobar and segmental lung regions analyzed in this work and are unlikely to substantively affect the results and conclusions. Finally, for legacy reasons this study was performed with hyperpolarized ^3^He gas, while hyperpolarized 129Xe gas is far more common in clinical research at present. We anticipate a similar functional relationship between VDP, fSAD, and airway mucus using HP ^129^Xe MRI (McIntosh et al., 2022). Studies directly comparing the two gases in the same patients show qualitatively larger VDP using HP ^129^Xe MRI presumably due to increased density of the gas mixture (Kirby et al., 2013). It is therefore conceivable that VDP measured with HP 129Xe MRI will have improved sensitivity to obstruction compared to fSAD, but testing of this hypothesis is left to future studies.

## 5 Conclusion

In conclusion, areas of ventilation defect as found on HP ^3^He MRI in asthma are spatially and globally correlated with areas of fSAD on CT, as determined using PRM. On the segmental level, our model predicting fSAD showed a quadratic relationship with VDP, and potentially improved sensitivity to mucus plugs for fSAD when obstruction is mild, i.e., sVDP is within 4%–10%. Overall, mucus plugs were more consistently associated with large sVDP than with large fSAD in the corresponding airway segment, possibly because of the larger response from the absence of airways distal to the obstruction.

## Data Availability

The original contributions presented in the study are included in the article/Supplementary Materials, further inquiries can be directed to the corresponding author.

## Abbreviations

%P, Percent predicted; BD, Bronchodilator; BMI, Body mass index; COPD, Chronic obstructive pulmonary disease; CT, Computed Tomography; FEV_1_, Forced expiratory volume in one second; FRC, Functional residual capacity; fSAD, Functional small airways disease; FVC, Forced vital capacity; HP ^3^He MRI, Hyperpolarized helium-3 magnetic resonance imaging; PRM, Parametric Response Mapping; sVDP, segmental VDP; VDP, Ventilation defect percent.
