# Supplementary figures and images for: Comparison of hyperpolarized 3He-MRI, CT based parametric response mapping, and mucus scores in asthmatics
Source: Front Physiol. 2023 Aug 1;14:1178339. doi: 10.3389/fphys.2023.1178339 (PMC10431597; doi:10.3389/fphys.2023.1178339)

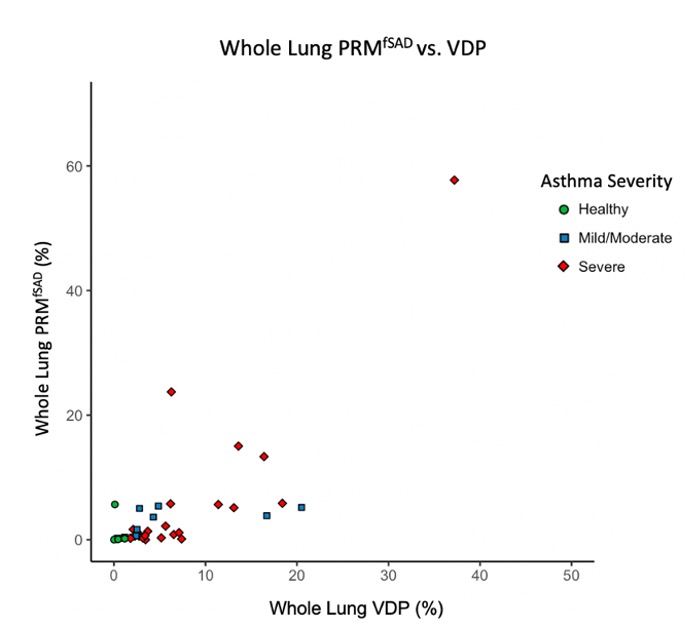

Supplement: Supplementary file 1 [file Image1.JPEG]
