# Supplementary material for: Comparison of hyperpolarized 3He-MRI, CT based parametric response mapping, and mucus scores in asthmatics
Source: Front Physiol. 2023 Aug 1;14:1178339. doi: 10.3389/fphys.2023.1178339 (PMC10431597; doi:10.3389/fphys.2023.1178339)
Supplement: Supplementary file 2 [file DataSheet1.pdf]

Appendix A – Scan Parameters

CT Parameter	64 Detector CT
Collimation	64 X 0.625 mm
Rotation Time (s)	0.5
Pitch	0.984
Effective mAs	100 - 180
Peak Kilovoltage (kVp)	120
Matrix	512 x 512
Acquired slice thickness (mm)	0.625
Interval (mm)	0.5
Reconstruction Algorithm	Standard

Table S1: *CT imaging acquisition parameters for both inspiratory and expiratory CTs obtained in this study.*

MRI Parameter	HP ^{3}He MRI	Matching Proton MRI
Pulse sequence	Fast 2D gradient echo	2D SSFSE
TR/TE	6.5/2.9 ms	450/37ms
Flip angle	14°	90°
Matrix	128 x 128 in-slice	128 x 128 in-slice
FOV	40 cm	40 cm
Reconstructed voxel dimension	1.56 x 1.56 x 15 mm	1.56 x 1.56 x 15 mm
Slices	Sufficient to cover lung volume	Sufficient to cover lung volume

Table S2: *MRI acquisition parameters for both HP ^{3}He MRI and the matching proton MRI that were acquired.* ***Definition of abbreviations:*** *TR – repetition time, TE – echo time, FOV – field of view.*

	VDP	Mucus Score	FEV1 PP	FVC PP	FEV1/FVC PP	FEF_{25-75}PP
**VDP**	--	0.68 ***	-0.60 ***	-0.38 *	-0.60 ***	-0.58**
**fSAD**	0.65 ***	0.55 **	-0.56 ***	-0.35 *	-0.59 ***	-0.57***
**Emph**	0.43 **	0.44	-0.30	-0.07	-0.45 **	-0.36*

Table S3: *Spearman correlation coefficients for whole lung VDP, and PRMfSAD, and PRMemph (listed on left) when compared to VDP, mucus score, and spirometry. PP=Percent Predicted. Significance Key: *p<0.05, **p<0.01, ***p<0.001.*

Appendix B – Statistical Model Details

The model of best fit for comparing segmental fSAD as a function of VDP was a piecewise quadratic model. The model is fit to all segmental data while correcting for multiple data points from each segment. All segments fit to the same model, but each has a different intercept, or shift. The equations for the non-zero VDP piecewise models are given below. In both equations, B is a segment specific shift that differs between bronchopulmonary segments, but is the same value for both equations within the same segment.

Piecewise equation for VDP>0 and absence of mucus plug:

$$\log_{10} PRM_{fSAD} = 0.14(\log_{10} VDP)^2 + 0.29(\log_{10} VDP) + B$$

Piecewise equation for VDP>0 and presence of mucus plug:

$$\log_{10} PRM_{fSAD} = 0.21(\log_{10} VDP)^2 - 0.73(\log_{10} VDP) + 0.74 + B$$

Data used in this analysis are from the Severe Asthma Research Program and can be made publicly accessible upon request to the corresponding author (https://sarp.hmc.psu.edu).
